# Paediatric Choroidal Neovascularisation of Unknown Cause

**DOI:** 10.7759/cureus.78381

**Published:** 2025-02-02

**Authors:** Tasneem Elghazali Bakhiet, Shoaib Hassan, Jai Shankar

**Affiliations:** 1 Ophthalmology, Cardiff and Vale University Hospital, Cardiff, GBR; 2 Ophthalmology, Wrexham Maelor Hospital, Wrexham, GBR

**Keywords:** anti-vegf therapy, choroidal neovascularisation, emergency paediatric ophthalmology, oct (optical coherence tomography), subretinal fluid

## Abstract

Choroidal neovascularisation (CNV) is a condition characterised by the proliferation of abnormal blood vessels within the choroid. These vessels tend to leak blood and fluid into the adjacent tissues, thereby causing harm to the retina and posing a threat to visual function. The infrequency of CNV in children can be attributed to its predominant association with age-related macular degeneration (AMD). The incidence of CNV in paediatric patients is generally considered to be very low, with rates often cited as being less than one case per 100,000 individuals annually.

We present a 10-year-old female who was referred by an optician due to a one-month history of unilateral blurred vision in her left eye, leading to difficulties in reading the school board. The patient had no other past medical, trauma, or ocular history, and her family and developmental history were non-significant.

On examination, the patient’s visual acuity measured 6/6 in the right eye and 6/9 in the left eye. Initial dilated fundal examination revealed a yellow hypopigmented, raised subfoveal lesion with irregular borders in her left eye; however, there was no evidence of neovascularisation or haemorrhage. The patient presented two weeks later with a sudden visual drop of 6/60. Fundal examination showed a new haemorrhage in the left eye, and subretinal fluid (SRF) was noted within the macula. Optical coherence tomography (OCT) and OCT angiography (OCT-A) identified an increase in SRF and confirmed a solitary CNV. The child received three loading doses of intravitreal anti-vascular endothelial growth factor (anti-VEGF) at one-month intervals, leading to complete resolution of SRF and haemorrhage on OCT. Consequently, the patient’s visual acuity improved to 6/12.

In summary, idiopathic CNV, although rare in paediatric patients, must be promptly diagnosed and adequately treated to guarantee resolution.

## Introduction

Choroidal neovascularisation (CNV) is a feature of macular degeneration characterised by the growth of abnormal blood vessels in the choroid, the middle layer of the eye situated between the sclera and the retina. These vessels can leak fluid and blood, damaging the retina and leading to central vision loss [1].

CNV is histologically classified into three main types [2]:

(1) Occult CNV: neovascularisation confined between the retinal pigment epithelium (RPE) and Bruch’s membrane, originating from the choroid.

(2) Classic CNV: neovascularisation arising from the choroid that penetrates the RPE into the subretinal space.

(3) Retinal angiomatous proliferation (RAP): neovascularisation originating within the neurosensory retina that progresses posteriorly into the subretinal space.

CNV is particularly rare in children, with an estimated annual incidence of 0.21 per 100,000 in the UK [3]. The largest study on paediatric CNV to date analysed the records of 4,883,839 patients under 18 years of age in the Intelligence Research in Sight (IRIS) registry over a five-year period. It identified 2,353 eyes of 1,920 patients (0.04%) with a diagnosis of CNV [4]. This rarity is primarily because CNV is often associated with age-related macular degeneration (AMD), a condition not seen in children [1].

Additionally, children typically have a healthier and more robust choroid and RPE, coupled with stronger vascular integrity and a more efficient immune response compared to adults. These factors provide protection against the pathological angiogenesis that underlies CNV, significantly reducing the likelihood of its occurrence in the paediatric population [1].

We present a rare case of idiopathic solitary CNV in a paediatric patient.

## Case presentation

A previously healthy 10-year-old girl was referred to the ophthalmology clinic by her optician due to blurred vision in her left eye. The symptom had persisted for over a month, affecting her ability to see the board at school. Her right eye was unaffected. The patient had no significant past ocular or medical history. Notably, she experienced a suspected seizure three months earlier, which was assessed by a paediatric team and attributed to vasovagal syncope before discharge.

Her birth history was uneventful, with a normal pregnancy, vaginal delivery, and developmental milestones. Her family and social history were unremarkable, with no known genetic conditions, and she had five healthy siblings.

Examination

**Figure 1:**
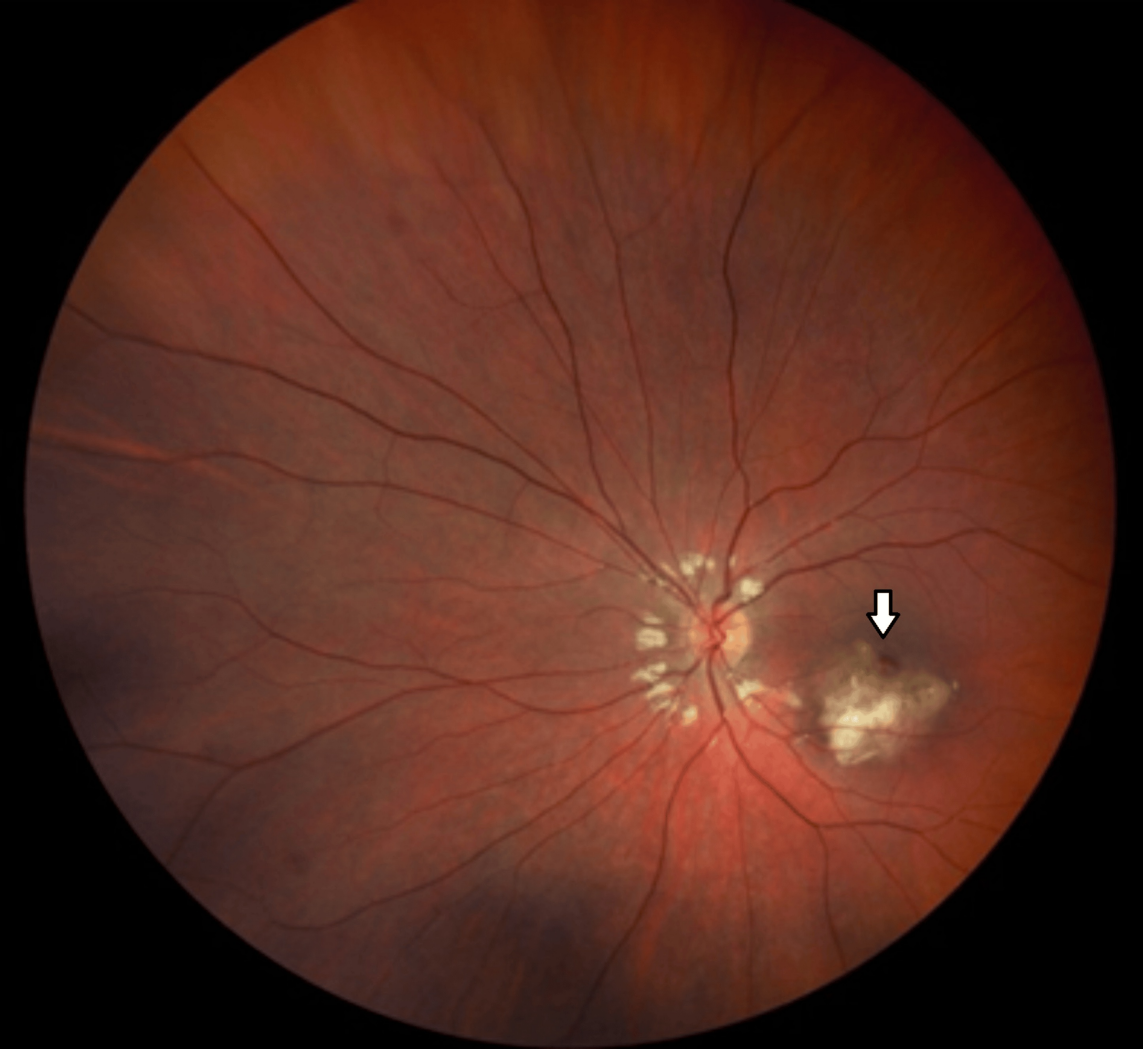
Fundoscopy of the left eye showing yellow hypopigmented, raised subfoveal lesion with irregular borders (arrow)

On initial examination, her visual acuity was 6/6 in the right eye and 6/9 in the left eye. Pupillary responses were normal, with no evidence of a relative afferent pupillary defect (RAPD). The anterior segment examination was unremarkable. A dilated fundus examination showed a white-to-yellow hypopigmented, raised subfoveal lesion with irregular borders in the left eye, with no associated haemorrhage or neovascularisation (Figure 1). The right eye was normal.

**Figure 2:**
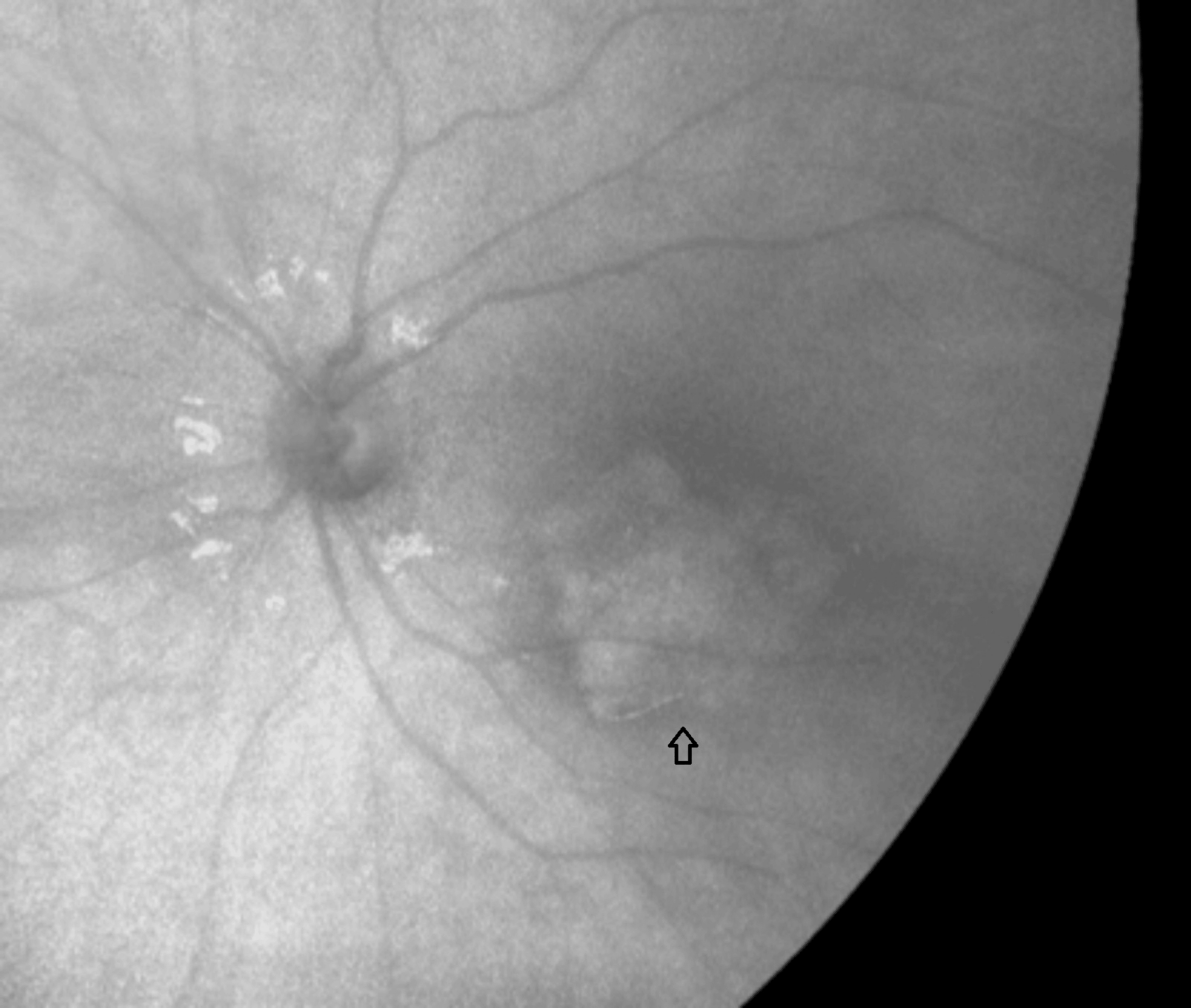
Fundus autofluorescence of the left eye showing hypo-autofluorescence in the subfoveal region

**Figure 3:**
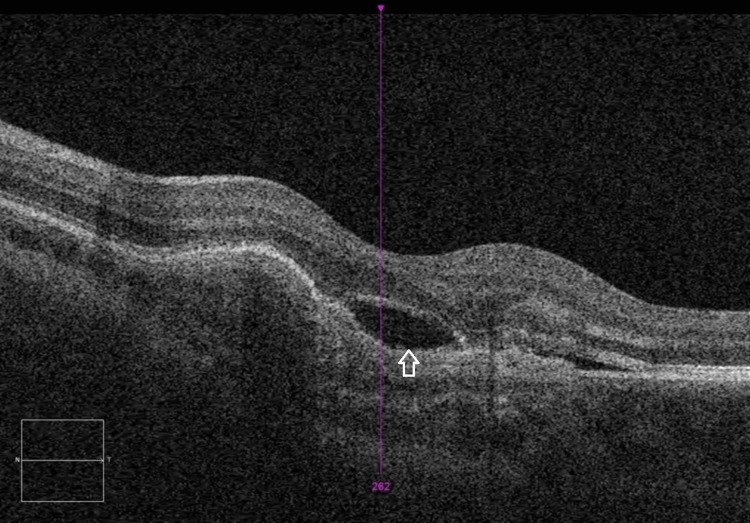
OCT of the left eye showing disruption of the RPE layer at the macula and fovea and SRF (arrow) OCT, optical coherence tomography; RPE, retinal pigment epithelium; SRF, subretinal fluid

She underwent fundus photography and autofluorescence, which revealed hypo-autofluorescence in the subfoveal region (Figure 2). Optical coherence tomography (OCT) of the macula demonstrated disruption of the RPE layer at the macula and fovea, along with some subretinal fluid (SRF), with a central macular thickness (CMT) of 311 µm (Figure 3). Blood tests, including inflammatory markers and toxoplasmosis serology, were normal.

**Figure 4:**
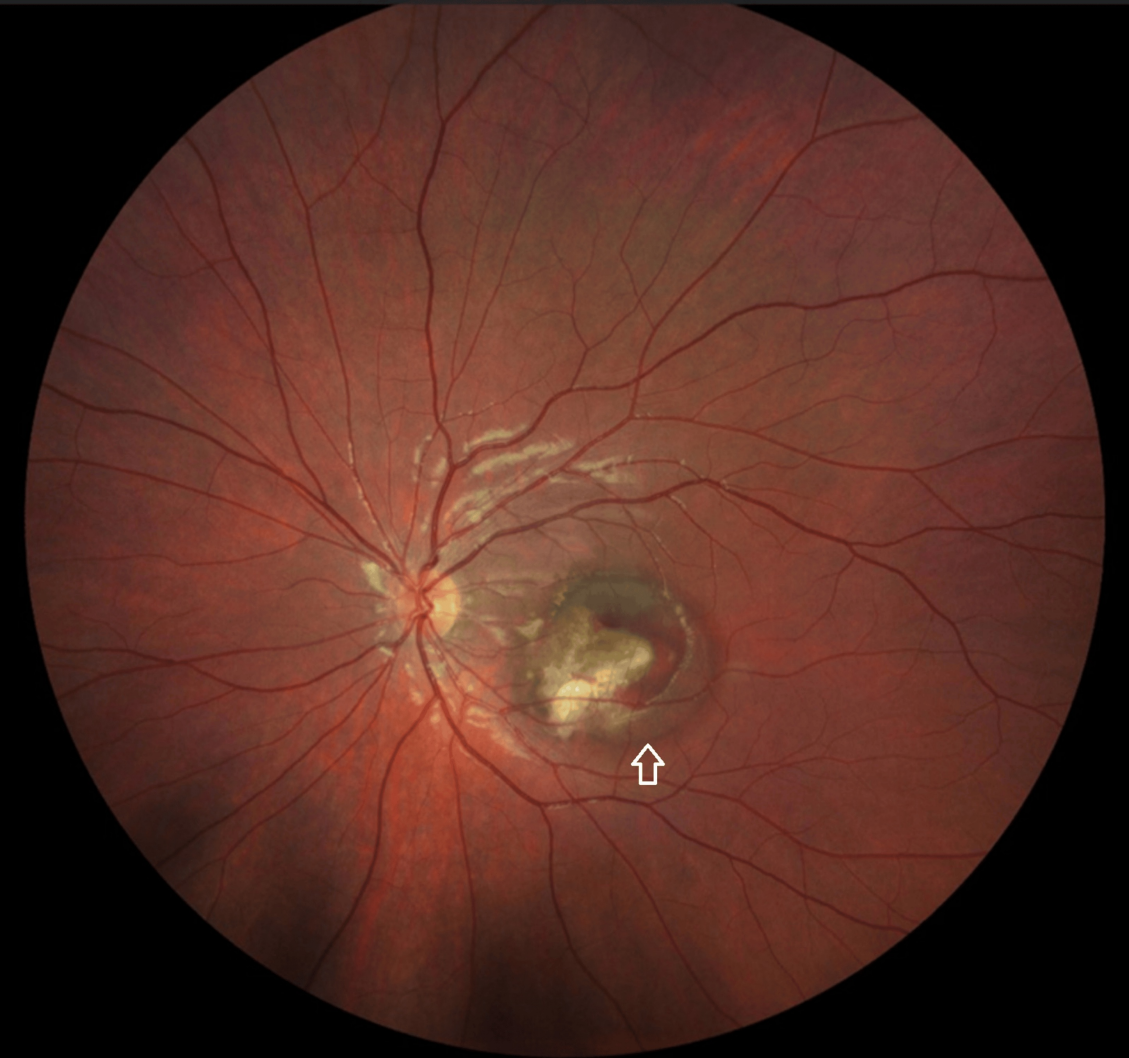
Fundoscopy of the left eye showing subretinal haemorrhage and SRF at the macula SRF, subretinal fluid

At her two-week follow-up, her vision in the left eye had declined sharply to 6/60. Repeat fundus examination revealed the development of subretinal haemorrhage and SRF at the macula (Figure 4).

**Figure 5:**
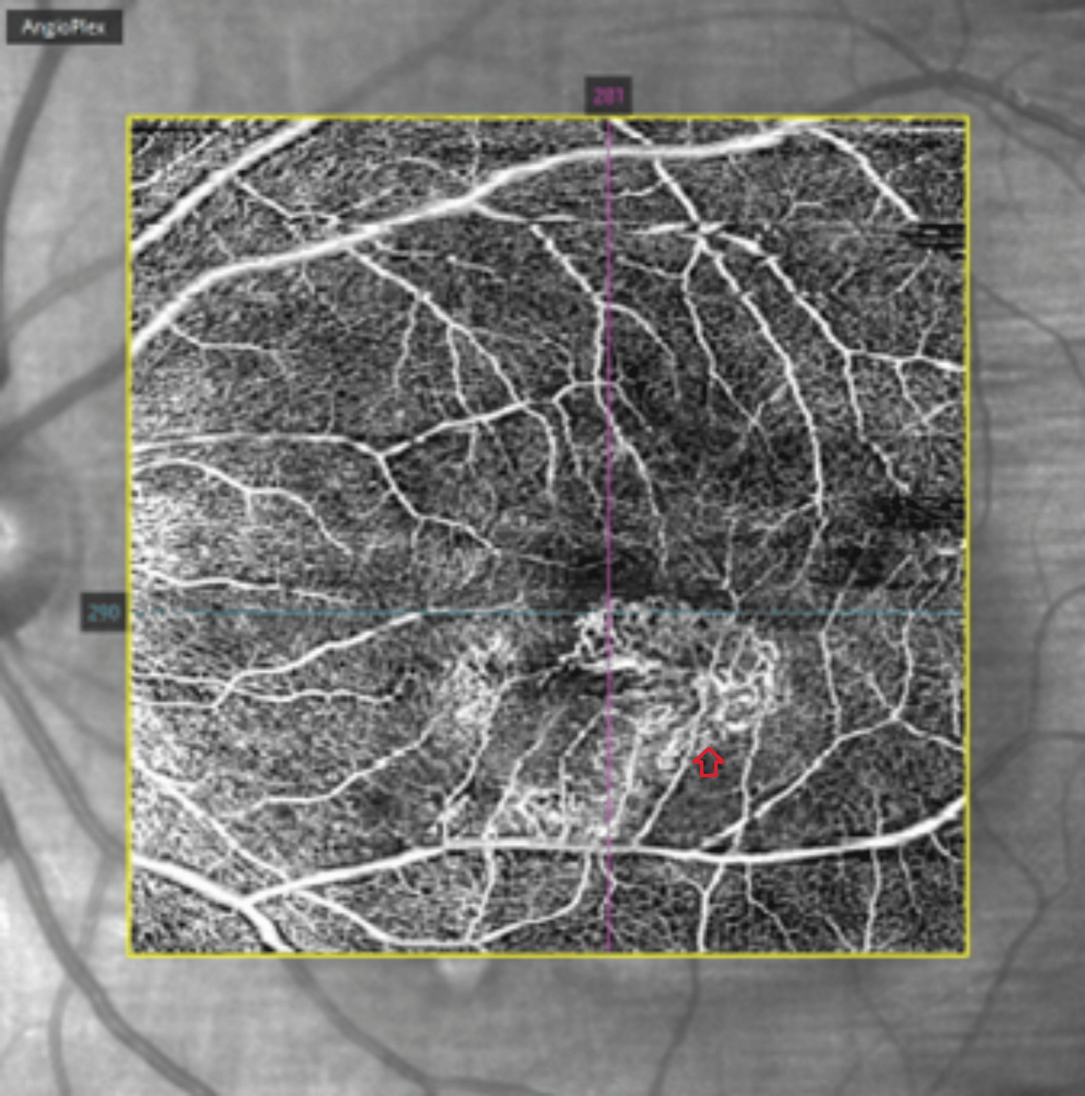
OCT-A of the left eye showing solitary choroidal neovascularisation OCT-A, optical coherence tomography angiography

OCT imaging showed increased SRF and RPE thickening. OCT angiography (OCT-A) identified a solitary choroidal neovascular membrane (Figure 5).

Treatment and outcome

**Figure 6:**
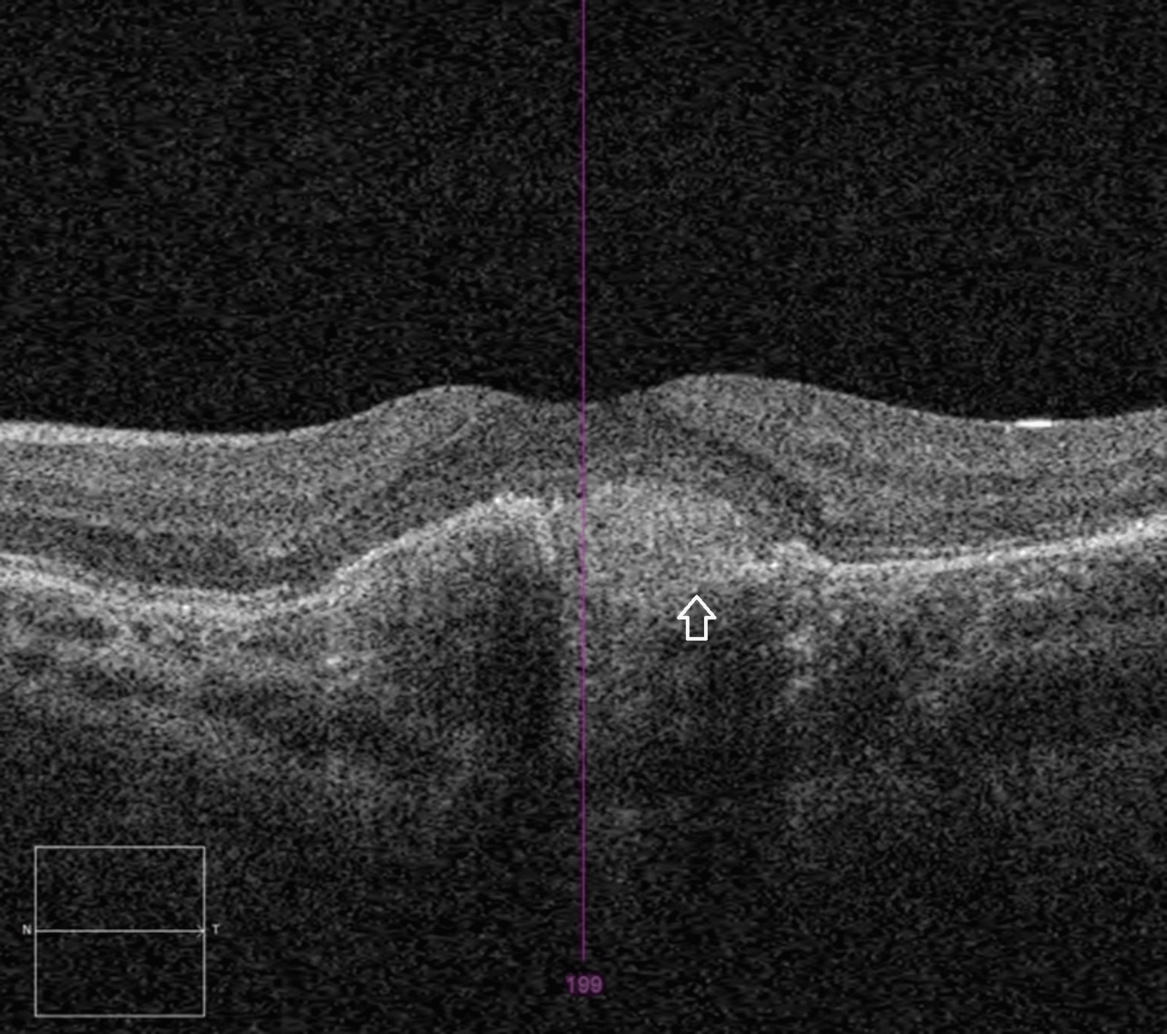
OCT of the left eye post-anti-VEGF treatment showing resolution of subretinal fluid OCT, optical coherence tomography; VEGF, vascular endothelial growth factor

The patient was diagnosed with idiopathic CNV. She was referred to a tertiary centre for further management. She received three monthly intravitreal injections of anti-vascular endothelial growth factor (anti-VEGF) (ranibizumab). Significant improvement was noted after the first injection, with clinical resolution of subretinal haemorrhage and SRF on OCT (Figure 6). By the end of the treatment course, her visual acuity improved to 6/12 in the left eye.

## Discussion

CNV in paediatric cases is rare but remains a significant cause of central vision loss. Key differences between children and adults include the rarity of macular degeneration and myopic fundus in younger individuals, the absence of calcification and thickening of Bruch’s membrane, and the frequent presence of a solitary subretinal in-growth, typically located in the subfoveal region [5]. The most common histological type observed is classic CNV [5].

In paediatric cases, it is crucial to identify and address underlying aetiology, which may include post-inflammatory conditions (e.g., choroiditis), infectious causes (e.g., toxoplasmosis), and hereditary retinal dystrophies like Best’s disease. However, many cases remain idiopathic [6]. This is evident in a case involving a solitary subretinal CNV of unknown cause.

Diagnosing CNV in children presents significant challenges, as the gold standard, fluorescein angiography (FA), is an invasive and often uncomfortable procedure for both the patient and the clinician. However, OCT-A has emerged as a valuable, non-invasive diagnostic alternative [7]. Ong et al. demonstrated that OCT-A can effectively differentiate between active and quiescent CNV [7]. Active CNV is characterised by dense, fine capillaries with frequent anastomoses and vascular loops, which resolve following treatment. In contrast, quiescent CNV appears as larger, mature vessels with lower capillary density and lacks the anastomoses and loops seen in active CNV. These findings support the use of OCT-A as a child-friendly alternative to FA, reducing the need for invasive procedures such as those requiring general anaesthesia in paediatric patients [7]. Furthermore, oral FA serves as an effective alternative when intravenous access is challenging in paediatric patients. Conner et al. demonstrated that oral FA combined with ultrawide-field imaging offers valuable clinical insights and is well-tolerated by children [8]. A standardised dose of 4 mL of 25% fluorescein is recommended for children over three years of age and weighing more than 20 kg [8].

Management of paediatric CNV varies based on the underlying cause and clinical presentation. While some cases may spontaneously regress and require only observation, treatment often involves photodynamic therapy (PDT) or intravitreal anti-VEGF injections [6]. Paediatric CNV has a higher likelihood of spontaneous regression compared to adult cases, attributed to the relatively intact RPE-Bruch’s membrane complex in children. However, despite the potential for spontaneous resolution, studies have consistently shown that treated eyes achieve superior visual outcomes [6].

Kozak et al. reported that 48% of paediatric patients with CNV experienced a gain of more than three lines of vision after intravitreal anti-VEGF therapy, with 60% achieving a final visual acuity of 20/50 or better [9]. This underscores the importance of early diagnosis and timely intervention to optimise visual outcomes.

Although no standardised treatment guidelines currently exist for paediatric CNV, anti-VEGF therapy has emerged as the most effective and retina-sparing option. Compared to other modalities, it is less destructive to the retina and RPE [6]. Children also tend to respond more favourably to anti-VEGF therapy than adults, often requiring fewer injections [10]. Padhi et al. found that an average of 2.11 anti-VEGF injections were sufficient for CNV regression or stabilisation, with 20% of cases achieving stabilisation after a single injection [10]. This aligns with our case, where significant improvement was observed following the first anti-VEGF injection.

These findings highlight the need for vigilance in diagnosing paediatric CNV and the efficacy of anti-VEGF therapy as the first-line treatment.

## Conclusions

This case underscores the critical importance of early recognition and timely treatment of paediatric subfoveal lesions associated with CNV. Although standardised guidelines for managing paediatric CNV are lacking, the significant visual improvement observed with anti-VEGF therapy highlights its efficacy as a primary treatment option for children. Additionally, the unique challenges of diagnosing CNV in paediatric patients emphasise the need to employ alternative, child-friendly diagnostic approaches to ensure accuracy and tolerability.

## References

[1] Kesen MR, Cousins SW (2010). Choroidal neovascularization. Encyclopedia of the Eye.

[2] (2024). Choroidal Neovascularization: OCT angiography findings. https://eyewiki.org/Choroidal_Neovascularization:_OCT_Angiography_Findings.

[3] Moosajee M, Abbouda A, Foot B, Bunce C, Moore AT, Acheson J (2018). Active surveillance of choroidal neovascularisation in children: incidence, aetiology and management findings from a national study in the UK. Br J Ophthalmol.

[4] Finn AP, Fujino D, Lum F, Rao P (2022). Etiology, treatment patterns, and outcomes for choroidal neovascularization in the paediatric population: an Intelligent Research in Sight (IRIS®) Registry Study. Ophthalmol Retina.

[5] Zhang T, Wang Y, Yan W (2021). Choroidal neovascularization in pediatric patients: analysis of etiologic factors, clinical characteristics and treatment outcomes. Front Med (Lausanne).

[6] Sobol EK, Hubbard GB (2023). Pediatric choroidal neovascularization. Pediatric Vitreoretinal Surgery.

[7] Ong SS, Hsu ST, Grewal D, Arevalo JF, El-Dairi MA, Toth CA, Vajzovic L (2020). Appearance of pediatric choroidal neovascular membranes on optical coherence tomography angiography. Graefes Arch Clin Exp Ophthalmol.

[8] Conner EA, Eldib A, Hiasat JG (2023). Pediatric oral fluorescein angiography: a retrospective review from a single institution. J Am Assoc Pediatr Ophthalmol Strabismus.

[9] Kozak I, Mansour A, Diaz RI (2014). Outcomes of treatment of pediatric choroidal neovascularization with intravitreal antiangiogenic agents: the results of the KKESH International Collaborative Retina Study Group. Retina.

[10] Padhi TR, Anderson BJ, Abbey AM (2018). Choroidal neovascular membrane in paediatric patients: clinical characteristics and outcomes. Br J Ophthalmol.

